# Duplex ultrasound and pedal acceleration time as tools to evaluate foot perfusion: a literature review

**DOI:** 10.1590/1677-5449.202300172

**Published:** 2024-03-25

**Authors:** Drako de Amorim Souza, Pedro Victor Freitas Medrado, Vinícius Alves Santos, Carolline Xavier de Aguiar, Guilherme Souza Silva, Lucas Pereira Pintos de Sousa, Yasmin Bione Diniz Amando, Paulo Fernandes Saad

**Affiliations:** 1 Universidade Federal do Vale do São Francisco - UNIVASF, Petrolina, PE, Brasil.; 2 Universidade Federal do Vale do São Francisco - UNIVASF, Hospital Universitário - HU, Petrolina, PE, Brasil.

**Keywords:** ultrasonography, duplex, peripheral arterial disease, foot, ultrassonografia duplex, doença arterial periférica, pé

## Abstract

Currently, the standard non-invasive test for diagnosing Peripheral Arterial Disease is the Ankle-Brachial Index. However, this test becomes unfeasible in a certain population. New evidence proposes the pedal acceleration time, an ultrasound index, as an alternative test. An integrative bibliographic review was carried out between June 3, 2022 and January 8, 2023, to investigate this new index as a tool to assess foot perfusion. Papers published in English, Portuguese, or Spanish between 2012 and 2022 were searched on PubMed, Google Scholar, and Scielo, using the keywords “Peripheral Arterial Disease” AND “Acceleration Time” AND (Pedal OR Plantar). Research that didn’t assess foot perfusion using the methods of interest or did not present human data and also case series or reports were excluded. Seven out of the sixty-six articles identified in the searches were selected for the review, all of which had notable methodological limitations. Pedal acceleration time seems to be able to diagnose and stratify and may reflect prognosis.

## Resumo

Atualmente, o teste não invasivo padrão para diagnosticar a doença arterial periférica é o índice tornozelo-braquial, porém, em uma determinada população, este torna-se inviável. Novas evidências propõem o tempo de aceleração plantar, que é um índice ultrassonográfico, como alternativa. Para investigá-lo no contexto do estudo da perfusão do pé, uma revisão bibliográfica integrativa foi realizada entre 03/06/2022 e 08/01/2023. Artigos em inglês, português ou espanhol, publicados entre 2012 e 2022 foram pesquisados através das ferramentas PubMed, Google Scholar e SciELO, com as palavras-chave “Peripheral Arterial Disease” AND “Acceleration Time” AND (Pedal OR Plantar). Pesquisas que não avaliaram a perfusão do pé pelos métodos de interesse, sem dados em humanos e relatos ou séries de casos, foram excluídos. Dos 66 artigos, 7 foram selecionados; todos com notáveis limitações metodológicas. O tempo de aceleração plantar aparenta ser capaz de diagnosticar, estratificar e prognosticar os doentes.

## INTRODUCTION

Peripheral arterial disease (PAD) is a prevalent and highly morbid disease caused by an atherosclerotic process that obstructs arterial blood flow and may reduce perfusion downstream of the obstruction. It is one of the cardiovascular manifestations of atherosclerosis, which forms a spectrum of presentations along with ischemic coronary disease and ischemic stroke. In PAD, the atheromatous obstruction most commonly affects the lower limbs.^1^

Although underdiagnosed, PAD has a high prevalence worldwide, with estimated rates in the population above 10% and close to 30% in patients aged 50 years or older.^2^ Unfortunately, a portion of this population evolves to chronic limb-threatening ischemia (CLTI), a complication of PAD that involves risk of amputation, impacts quality of life, and can lead to death.^3^

Non-invasive methods for studying arterial anatomy and function are fundamental for assessing patients with PAD, especially as a screening tool, since they are associated with fewer complications than invasive procedures. The standard method used for this purpose is the ankle-brachial index (ABI). However, it is known to have low accuracy in a subset of patients, particularly in patients with diabetes and chronic renal failure, and it reflects perfusion at the ankle level.^4,5^

A new ultrasound measurement, called the pedal acceleration time (PAT), offers promising results in cases not covered by the previous method.^4^ It is an index that is determined using duplex ultrasound (DUS) to measure the acceleration time (in milliseconds) at the arteries of the feet.^6^ It appears to be a tool with potential for analysis of peripheral perfusion in patients with non-compressible vessels and seems to provide valuable information concerning diagnosis, follow-up, intervention, and evaluation of wound healing according to blood flow to the angiosomes of the foot.^6,7^

This review aims to synthesize and critique the evidence on DUS and PAT as tools to assess foot perfusion from the perspective of PAD diagnosis and prognosis.

## METHODS

An integrative bibliographic review was conducted between June 3, 2022, and January 8, 2023. Searches were run using the descriptors “Peripheral Arterial Disease” AND “Acceleration Time” AND (Pedal OR Plantar) on the following search engines: PubMed, Google Scholar, and Scielo. We limited the results to articles published from 2012 to 2022 that presented those descriptors in any part of the text. Articles were selected for abstract review based on title and matched descriptors as long as they discussed the review subject: DUS applied to the arteries of the foot using the acceleration time index.

Subsequently, abstracts were read to assess inclusion and exclusion criteria. Due to the lack of articles on the subject, retrospective, prospective, and cross-sectional observational studies in Spanish, English, or Portuguese were included. After reading abstracts and entire papers, we excluded research that deviated from the theme of this review (i.e., duplex ultrasonography applied to the arteries of the foot using acceleration time index) or that did not present primary data on humans (e.g., reviews and laboratory studies) and also excluded case reports and case series.

After extensive reading, the studies were classified into two categories: diagnosis and correlation with other indices or prognostic studies. They were compared with each other to check for incongruities, common limitations, or methodological weaknesses, which were discussed with the group of researchers. No systematic method was used to investigate the limitations.

The studies included in the review are presented and summarized in Table 1, which lists the type of study, results, and level of evidence (LE), classified according to the Clinical Information Access Portal (CIAP), as shown in Table 2.^13^

**Table 1:** Review results.

**Author**	**Study type (duration)**	**Sample (limbs)**	**Results**	**LE**
Trihan et al.^8^	Cross-sectional study with consecutive patients (12 months)	77 (88)	● Significant linear correlation (R = -0.46, P < 0.01) between ATmax and ABI;	II
			● Significant linear (R = -0.78, P < 0.0001) and multivariate correlation between ATmax and TBI;	
			● ATmax ≥ 215ms diagnoses critical ischemia according to toe pressure (TP <= 30) with S = 85.7 (57.2-98.2), E = 81.1 (70.3-89.3), AUC = 0.89 (0.81-0.98), and 95%CI.	
Sommerset et al.^6^	Cross-sectional study (12 months)	250 (499)	● Significant linear correlation (p<0.001) between lateral plantar artery PAT and ABI;	III
			● Class 1 (no symptoms, ABI 0.90-1.30) correlated with PAT 89.9 ± 15.5 ms, Class 2 (moderate claudication, ABI 0.69-0.89) correlated with PAT 152.3 ± 28.4 ms, Class 3 (severe claudication, ABI 0.40 -0.68) correlated with PAT 209.8 ± 28.4 ms and Class 4 (critical ischemia, ABI 0.39-0.00) correlated with PAT 270.2 ± 35.3 ms;	
Teso et al.^4^	Retrospective cohort (12 months)	72 (73)	● Post-revascularization PAT in the range of 213 ± 47 ms and difference between pre- and post-revascularization PAT in the range of 30 ± 30 ms correlated with significant amputation (p < 0.00001);	III
			● Post-revascularization PAT in the range of 122 ± 30 ms and difference between pre- and post-revascularization PAT in the range of 117 ± 34 ms correlated with limb salvage (p < 0.00001);	
			● There was no significant difference between the amputation and limb salvage groups with respect to pre-revascularization PAT (p = 0.54);	
Santos^9^	Cross-sectional study (10 months)	141 (198)	● Significant Spearman correlation (ρ) between PAT and ABI in diabetics: ρ = -0.8016 (-0.8627 to -0.7174 95%CI; p<0.001); non-diabetics: ρ = -0.8071 (-0.8693 to -0.7198 95%CI; p<0.001); men: ρ = -0.8133 (-0.8693 to -0.7368 95%CI; p<0.001) and women: ρ = -0.7611 (-0.8397 to -0.6511 95%CI; p<0.01);	III
			● PAT values to estimate the degree of ischemia were calculated, notably a PAT > 196 ms indicates grade 3 ischemia (ABI < 0.4) in diabetics with S = 0.88, E = 0.87 and Ac = 0.88 and in non-diabetics with S = 0.83, E = 0.98 and Ac = 0.87;	
			● PAT was associated with moderate to high risk of amputation by the SVS-WIfI system, with a value > 145 ms in diabetics having S = 0.77, E = 0.77 and Ac = 0.77 and a value > 165 ms in non-diabetics having S = 0.78, E = 0.79 and Ac = 0.79;	
Geskin et al.^10^	Cross-sectional study (5 months)	30 (38)	● There was a significant reduction in mean PAT after revascularization (193 ± 118.41 ms to 160.38 ± 92.19 ms, p = 0.009);	III
			● Negative Pearson correlation between PAT and ABI before revascularization (r2 = 0.41, p = 0.002, n = 21);	
Ochoa-Ayón et al.^11^	Prospective cohort (1 year)	31 (31)	● After angioplasty, all patients (n = 31) had a significant reduction in PAT (mean of 213.00 ± 83.26 ms before and 118.35 ± 16.40 ms after angioplasty, p = 0.000);	III
			● After 1 year, the mean time to wound healing was 4.35 ± 2.42. Only 1 patient did not achieve wound healing;	
			● There were no significant amputations (at or above the ankle). 51.6% of patients (n = 16) had a minor amputation;	
Arévalo Zamora and Cifuentes González^12^	Cross-sectional study (12 months)	68 (130)	● Pearson correlation of -0.67 between ABI and PAT in the arcuate artery of the foot and -0.65 in the lateral plantar artery;	III
			● Association between ABI < 0.5 and PAT > 160 ms (S = 92.3% and E = 83.7%, AUC = 0.89 (95%CI (0.839-0.959);	
			● PAT < 120 ms correlated with normal ABI, PAT 120-160 ms correlated with ABI between 0.5-0.9.	

**Table 2:** Grading of evidence.

	**Level**	**Intervention**	**Diagnosis**	**Prognosis**	**Etiology**
Least biased	**I**	Systematic review of Level II studies	Systematic review of Level II studies	Systematic review of Level II studies	Systematic review of Level II studies
	**II**	Randomized Controlled Trial	Cross-sectional study among consecutive patients	Inception cohort study	Prospective cohort study
	**III**	One of the Following:	One of the following:	One of the following:	One of the following:
		- Non randomized experimental study (e.g. controlled pre- and post-test intervention study)	- Cross-sectional study among non-consecutive patients	- Untreated control patients in a randomized controlled trial	- Retrospective cohort study
		- Comparative (observational) study with a concurrent control group (e.g. cohort study, case-control study)	- Diagnostic case-control study	- Retrospectively assembled cohort study	- Case-control study
Most biased	**IV**	Case series	Case Series	Case series, or a cohort study of patients at different stages of disease	A cross-sectional study

Source: Clinical Information Access Portal (CIAP).^13^

## RESULTS

The search results identified 66, 12, and 1 articles from Google Scholar, PubMed, and Scielo, respectively. Of these, 36 were accessed after reading the title and keywords, 29 of which were excluded for the following reasons: reports or case series (3), no primary data on humans (11), and no relevance to the subject studied (15). In total, seven studies were selected: one retrospective cohort study, one prospective cohort study, and five cross-sectional studies, one of which one was a doctoral thesis and another of which was a specialization thesis. An analysis of the literature is shown in Table 1.

### Diagnosis and correlation with other indices

The first study^6^ to investigate the correlation between ABI and PAT was published in 2019. The expectation was that this tool could be used as an alternative option in cases of incompressible PAD. In a cross-sectional study, 250 non-diabetic patients and 499 lower limbs (LL) were studied, demonstrating a statistically significant linear correlation between ABI and PAT and significant differences between the mean PAT values for different clinical stratifications (from asymptomatic to critical ischemia). Notable limitations were as follows: a small number of patients were classified as having critical ischemia (cases with pain at rest and unhealed ischemic ulcer accounted for only 6% and 8% of the sample, respectively), which may affect the validity of the results in more severe cases of PAD; the sample did not represent diabetic or already revascularized patients; and the study did not report the strength of the correlation measured between PAT and ABI, or diagnostic test measures, such as sensitivity (S), specificity (E) and diagnostic accuracy. In common with the majority of publications studying PAT, this study did not clarify whether the sample size was sufficient. Also, the examinations were performed by several operators without considering inter-examiner variability.

Corroborating previous findings, a Brazilian cross-sectional doctoral thesis^9^ with 141 patients and 198 LL, including diabetics and non-diabetics classified as having CLTI and compressible ABI, demonstrated a strong Spearman correlation (ρ) between PAT and measured ABI (see Table 1). In addition, it was found that a PAT > 196 ms had accuracy (Ac) of around 87-88% for diagnosing critical ischemia according to the ABI criteria (critical ischemia defined as an ABI < 0.4) in both diabetic and non-diabetic patients. These results were significant for a sample of 153 LL or more, estimated with a power of 80% and a p-value of 0.05. Besides being a single-center study done by a single examiner, there were no other notable limitations.

A cross-sectional specialization thesis^12^ with 68 patients and 130 LL aimed to evaluate the correlation between PAT and ABI in a Colombian population. There were Pearson correlations of -0.67 and -0.65 between the ABI and the PAT of the arcuate and lateral plantar arteries of the foot, respectively. The study claims an association between the following values, but without further details: PAT > 160 ms with ABI < 0.5, PAT between 120 and 160 ms with ABI between 0.5 and 0.9, and PAT < 120 ms with normal ABI. Patients who were already revascularized or who had aortoiliac injuries were excluded. The study did not ensure statistical significance or specify whether the sample was statistically sufficient. Also, it did not specify whether the patients included were selected by convenience or give any details concerning the number of examiners or inter-examiner variability.

In a cross-sectional study^8^ with 77 consecutive patients and 88 lower limbs, which were non-revascularized and had no minor amputations (toe and more proximal), a correlation was found between the maximum acceleration time (ATmax: the greater value between the PAT of the dorsalis pedis artery or lateral plantar artery), ABI, and the toe-brachial index (TBI). Using the value of 30 mmHg of pressure at the big toe (TP: toe pressure) as a cutoff point to define critical ischemia, the study demonstrated that a maximum acceleration time ≥ 215 ms has good accuracy to diagnose critical ischemia, with sensitivity (S) = 85.7%, specificity (E) = 81.1%, and area under the curve (AUC) = 0.89. The main limitation of these results is the low sample size, especially in the subgroup of patients diagnosed with critical ischemia according to TBI (a total of 14 patients out of the 77 included, corresponding to 15.9% of the sample). Also, the study did not clarify whether the sample size was statistically sufficient.

Lastly, a cross-sectional study^10^ with 30 patients, 38 LL, and a duration of 5 months evaluated parameters for the study of micro and macro-circulation of the foot, including PAT. Among the results, 21 patients had compressible ABI and a significant Pearson correlation = -0.41 between PAT and ABI was found. Similar to other works on the subject, the study did not clarify whether the sample size is sufficient or whether the selection was by convenience or give any details concerning the number of examiners or inter-examiner variability.

### Prognosis

There is still scant evidence supporting PAT as a tool to stratify prognosis. In a retrospective cohort,^4^ the ability of PAT to predict successful revascularization at 1 year after the procedure in diabetic patients with CLTI was studied. Seventy-two patients were selected and limb salvage was shown to correlate with post-revascularization PAT in the range of 122 ± 30 ms or with a reduction of 117 ± 34 ms from the pre-revascularization value. However, the PAT measured before revascularization was not able to predict the success of the procedure. It was also possible to verify the range of values correlated with significant limb amputation (see Table 1). The study did not evaluate suprainguinal obstructions and infections with WIfI classes 2 and 3, which were excluded. As in many other studies on the subject, this study did not clarify whether the sample size was sufficient or whether sample selection was by convenience and did not provide any details concerning the number of examiners or inter-examiner variability.

These results were supported by those of a prospective cohort^11^ conducted with the objective of studying PAT as a predictive factor for limb salvage in patients diagnosed with CLTI undergoing angioplasty, excluding those who had suprainguinal lesions and those who had significant infection (WIfI classes 2 and 3). At 1 year, all of the 31 patients and 31 revascularized LL had a significant reduction in PAT (mean of 213.00 ± 83.26 ms before and 118.35 ± 16.40 ms after angioplasty, p = 0.000), with no significant amputations (at or above the ankle) recorded, although 51.6% of patients (n = 16) had a minor amputation. After 1 year, the mean time for wound healing was 4.35 ± 2.42 and only 1 patient did not achieve wound healing, but there was no statistical analysis to verify the statistical significance of these results or their correlation with PAT. It is concerning that the study had a small sample without a group with insignificant reductions in PAT (which was not clearly justified and could indicate selection bias). Other limitations, common to most studies in this review, were also present: the study did not clarify whether the sample size was sufficient or whether sample selection was by convenience and did not give any details concerning the number of examiners or inter-examiner variability.

A cross-sectional study^9^ already discussed in the previous section estimated an accuracy of 77% for PAT to predict the risk of amputation according to the SVS-WIfI classification. A PAT above 145 ms in diabetic patients and above 165 ms in non-diabetic patients were the best cutoff points for predicting moderate to high amputation risk, according to the SVS-WIfI classification. These results are limited because they do not directly assess the ability of PAT to measure amputation risk, but rather its association with the SVS-WIfI score, which is already well established in the literature.

## DISCUSSION

### Current perspectives on the ABI

Numerous non-invasive tools are used both for diagnostic purposes and to assess the severity of PAD and the most appropriate test varies depending on what is being elucidated.^14^ The importance of accurate diagnosis and assessment lies in the possibility of offering therapy to relieve symptoms and improve quality of life and of indicating revascularization in certain cases.^15^ The ABI is a first-line, non-invasive measure that is accessible, reproducible, inexpensive, and ideally indicated for all patients with a history or physical examination suggestive of PAD.^16-19^ Patients with claudication and an ABI < 0.9 have the diagnostic criteria for PAD.^12,14^

In some studies that evaluate its use by general practitioners, the test tends to be underused or performed incorrectly, due to incorrect technique.^20-22^ It is thought that time constraints, reimbursement, staff availability, and staff training are major factors that explain its underuse.^23^

The AHA/ACC guideline does not recommend ABI as a screening test in patients with a low pre-test probability of PAD,^18^ as there is insufficient evidence of benefit to support its use in asymptomatic adults as a screening method for PAD.^17,24^ Nevertheless, it is considered a predictor of cardiovascular risk by the Framingham score, in addition to being an important diagnostic tool for PAD and Critical Limb Ischemia (CLI).^25-27^

It is estimated that the ABI has a sensitivity of 61% (95%CI: 55-69) and specificity of 92% (95%CI: 89-95) for diagnosing stenosis greater than or equal to 50% of the arterial lumen.^28^ The inter and intra-examiner reliability of the ABI is acceptable, although there are inconsistencies in the methodology used to measure systolic blood pressure and calculate the ABI.^29^

However, the ITB has some significant limitations, the most common of which is the presence of incompressible vessels. Medial calcification, frequently observed in patients with diabetes, chronic kidney disease, and the elderly, causes overestimated ABI results.^6,10,12^ Thus, vascular stiffness due to calcific sclerosis decreases the diagnostic sensitivity of the disease with this method, resulting in an unreliable measurement for this group of patients and even making measurement impossible in some cases.^13,14^

Other alternatives to the ABI include the Toe-Brachial Index (TBI), physiological studies such as Segmental Perfusion Pressure, and Transcutaneous Oxygen Pressure (TcPO2), as well as imaging tests such as Duplex Ultrasound.^19^

### Other non-invasive tests

The TBI is an accepted method for diagnosing and assessing the severity of PAD, as well as a recognized modality for testing for small vessel disease.^5,8,23^ In the group most susceptible to presence of incompressible vessels, TBI has a much greater sensitivity than ABI and is therefore a preferable test for diabetics and other groups susceptible to arterial calcification.^23,28^ However, it is not an accessible tool and is not a viable option for health systems in developing countries.^8^ A preferable test for prognostic wound healing is TcPO2. In patients who have swelling or an extensive injury to the foot, measurements may give erroneous results, and they are generally unavailable in general centers.^4^ In turn, TP is another well-documented method for diagnosing PAD and predicting the healing of diabetic foot ulcers.^30^ Unfortunately, these tools are not available in many developing countries.^8,9^

In fact, except for the ABI, there are few options for non-invasive methods that enable the perfusion of the lower limbs to be studied and are accessible. It is necessary to develop an alternative method for this purpose, ideally to overcome its limitations. This is justified because, in addition to the limitations caused by arterial incompressibility,^31-33^ the ABI does not allow for the study of perfusion beyond the level of the ankle or provide informartion on the distal hemodynamics of the foot.^5^

### PAT as an alternative to the abi and other non-invasive tests

The study of the distal arteries of the foot using DUS, quantified in the PAT index, seems to be an alternative to ABI in cases in which it fails. Several recent studies have demonstrated a linear correlation between PAT and the ABI, when vessels are compressible, both in diabetics and non-diabetics,^6,8-10,12^ suggesting that PAT could be an alternative to ABI. Corroborating this hypothesis, one cross-sectional study verified a significant linear and multivariate correlation between the maximum PAT (ATmax) and the TBI,^8^ which is, as discussed, the preferable test in cases of incompressible vessels. Unfortunately, no similar studies were found to verify the reproducibility of their results.

Currently, evidence shows that PAT seems able to differentiate between degrees of clinical and ischemic severity, making it a potential tool to aid in the diagnosis of PAD and critical ischemia.^6,8,9^ A study^8^ involving severe stage PAD revealed that maximum acceleration time had high diagnostic accuracy for detecting critical limb threatening ischemia. It is possible that PAT could become an alternative for the definition of ischemia (classically defined using the ABI) used in the WIfI classification,^3^ due to the correlation between limb salvage and a pedal acceleration of less than 180 ms.^4^ However, evidence from studies of larger samples with statistically adequate sizes and randomization methodology are blind spots in the literature and should be considered before drawing further conclusions.

It is believed that PAT can help in definition of prognosis after revascularization, since one study^4^ demonstrated that a significant reduction in PAT after revascularization surgery correlated with a greater probability of saving the limb at 1 year. This result was supported by a similar but smaller study.^11^ However, none of those studies carried out a more detailed analysis of specific arteries linked to the perfusion of certain angiosomes in order to test the hypothesis that the evidence of improved flow documented by the PAT in a given artery correlates with a better response to the intervention. A third study^9^ supported these findings by demonstrating a significant correlation between PAT and the amputation risk score defined using the SVS-WIfI classification. Further studies are needed to assess the reproducibility of these findings, preferably with robust methodological designs. Other aspects of disease prognosis, such as the ability to predict wound healing time and quality of life, have yet to be demonstrated.

### Other future perspectives on the pat

The degree of peripheral tissue ischemia depends on the size and speed of formation of arterial stenosis, as well as the extent of collateral circulation. The network of collateral vessels guarantees a functional reserve for arterial irrigation. The greater the chronicity of the obstruction, the greater the collateral network.^34^ The angiosome concept was introduced in 1987^35^ to study the perfusion of regions of an anatomical portion. Defined as anatomical divisions of the arterial supply to areas of the foot, six pedal angiosomes are considered, three originating from the posterior tibial artery, one from the anterior tibial artery, and two from the fibular artery.^36^ The lateral plantar artery, a branch of the posterior tibial artery, is typically dominant and easily visualized with duplex, which justifies it being one of the main arteries to study using PAT.^6^

Because it involves the aspect of limb irrigation, this anatomical concept helps in understanding the restoration of blood flow in an ischemic injury.^37^ The arterial supply to the region of interest has a significant impact on predicting wound healing and on the success of the intervention itself.^6^ Quantitatively assessing the blood flow of a main artery that irrigates an angiosome and its collaterals should, therefore, help guide therapeutic choices in a similar way to the angiographic study, with the advantage of being non-invasive and being available at the bedside. However, this theory remains in the realm of speculation, since the current literature offers no evidence of PAT’s true diagnostic capacity for assessing the perfusion of specific segments by comparison with angiography.

### Limitations of the current literature

None of the studies published so far have robust designs, being observational analyses without rigorous methodology and subject to bias. Only one study^8^ specified how patients were recruited to the survey (by consecutive inclusion) and was classified as LE II. The vast majority did not specify sample analysis or ensure that the sample studied was statistically sufficient.^4,6,8,10-12^ None of the studies employed randomization of data or any type of blinding methodology.

Thus, since these limitations imply an important risk of bias, it is recommended that the results of other centers be evaluated and studies be designed that challenge the reproducibility of the findings, in order to estimate the true contribution that this new index can make to the specialty.

## CONCLUSIONS

DUS and PAT have shown promise for assessing foot perfusion through its main and collateral arteries. Despite several methodological limitations, studies have demonstrated a linear correlation between PAT and ABI, when vessels are compressible. PAT appears to be an alternative to the ABI, when vessels are incompressible. It seems to be capable of diagnosing and stratifying patients with PAD by degree of severity, in addition to having possible uses in defining disease prognosis, as a measure of success after revascularization, and for estimating the risk of amputation. However, the quantity and quality of studies published so far do not allow definitive conclusions to be drawn.

## References

[1] Brito CJ, Murilo R, Brito CJ, Silva RM, Araújo EL (2014). Cirurgia vascular - cirurgia endovascular, angiologia..

[2] Mascarenhas JV, Albayati MA, Shearman CP, Jude EB (2014). Peripheral arterial disease. Endocrinol Metab Clin North Am.

[3] Conte MS, Bradbury AW, Kolh P (2019). Global vascular guidelines on the management of chronic limb-threatening ischemia. J Vasc Surg.

[4] Teso D, Sommerset J, Dally M, Feliciano B, Vea Y, Jones RK (2021). Pedal Acceleration Time (PAT): a novel predictor of limb salvage. Ann Vasc Surg.

[5] Sommerset J, Teso D, Karmy-Jones R, Vea Y, Feliciano B (2020). Pedal flow hemodynamics in patients with chronic limb-threatening ischemia. J Vasc Ultrasound..

[6] Sommerset J, Karmy-Jones R, Dally M, Feliciano B, Vea Y, Teso D (2019). Plantar acceleration time: a novel technique to evaluate arterial flow to the foot. Ann Vasc Surg.

[7] Sommerset J, Teso D, Feliciano B (2019). Innovative arterial duplex examination: a guide to evaluate flow in the foot using pedal acceleration time. J Vasc Ultrasound..

[8] Trihan JE, Mahé G, Croquette M (2022). Accuracy of acceleration time of distal arteries to diagnose severe peripheral arterial disease. Front Cardiovasc Med.

[9] Santos GC (2021). Correlação entre o tempo de aceleração plantar, o índice tornozelo braquial e os escores SVS-WIfI em pacientes com isquemia crônica ameaçadora do membro.

[10] Geskin G, Mulock MD, Tomko NL, Dasta A, Gopalakrishnan S (2022). Effects of Lower limb revascularization on the microcirculation of the foot: a retrospective cohort study. Diagnostics.

[11] Ochoa-Ayón BL, Quiroz-Villegas ME, Valdovinos-Estrada JA (2022). Tiempo de aceleracion plantar como factor predictivo para salvamento de extremidad. Rev Mex Angiol.

[12] Arévalo Zamora C, Cifuentes González JC (2022). Utilidad de la medición del tiempo de aceleración plantar en el diagnostico de enfermedad arterial periférica.

[13] Clinical Information Access Portal (2016). Introduction to evidence-based practice and CIAP: grading levels of evidence.

[14] Harrington A, Kupinski AM (2022). Noninvasive studies for the peripheral artery disease patient. Semin Vasc Surg.

[15] Yagyu T, Funabashi S, Yoneda S, Noguchi T, Yasuda S (2020). Novel evaluation method for lower extremity peripheral artery disease with duplex ultrasound usefulness of acceleration time. Circ J.

[16] Danieluk A, Chlabicz S (2021). Automated measurements of ankle-brachial index: a narrative review. J Clin Med.

[17] Lin JS, Olson CM, Johnson ES, Whitlock EP (2013). The ankle-brachial index for peripheral artery disease screening and cardiovascular disease prediction among asymptomatic adults: a systematic evidence review for the U.S. preventive services task force. Ann Intern Med.

[18] Gerhard-Herman MD, Gornik HL, Barrett C (2017). 2016 AHA/ACC Guideline on the Management of Patients With Lower Extremity Peripheral Artery Disease: Executive Summary: A Report of the American College of Cardiology/American Heart Association Task Force on Clinical Practice Guidelines. Circulation.

[19] Criqui MH, Matsushita K, Aboyans V (2021). Lower extremity peripheral artery disease: contemporary epidemiology, management gaps, and future directions: a scientific statement from the American Heart Association. Circulation.

[20] Davies JH, Kenkre J, Williams EM (2014). Current utility of the ankle-brachial index (abi) in general practice: implications for its use in cardiovascular disease screening. BMC Fam Pract.

[21] Nexoe J, Damsbo B, Lund JO, Munck A (2012). Measurement of blood pressure, ankle blood pressure and calculation of ankle brachial index in general practice. Fam Pract.

[22] Hageman D, Pesser N, Gommans LNM (2018). Limited adherence to peripheral arterial disease guidelines and suboptimal Ankle Brachial Index Reliability in Dutch Primary Care. Eur J Vasc Endovasc Surg.

[23] Aboyans V, Criqui MH, Abraham P (2012). Measurement and interpretation of the Ankle-Brachial Index. Circulation.

[24] Curry SJ, Krist AH, Owens DK (2018). Screening for peripheral artery disease and cardiovascular disease risk assessment with the ankle-brachial index. JAMA.

[25] Fowkes G, Fowkes FGR, Murray GD (2008). Ankle brachial index combined with framingham risk score to predict cardiovascular events and mortality. JAMA.

[26] Jakubiak GK, Pawlas N, Cieślar G, Stanek A (2020). Chronic lower extremity ischemia and its association with the frailty syndrome in patients with diabetes. Int J Environ Res Public Health.

[27] Abouhamda A, Alturkstani M, Jan Y (2019). Lower sensitivity of ankle-brachial index measurements among people suffering with diabetes-associated vascular disorders: a systematic review. SAGE Open Med.

[28] Herráiz-Adillo Á, Cavero-Redondo I, Álvarez-Bueno C, Martínez-Vizcaíno V, Pozuelo-Carrascosa DP, Notario-Pacheco B (2017). The accuracy of an oscillometric ankle-brachial index in the diagnosis of lower limb peripheral arterial disease: a systematic review and meta-analysis. Int J Clin Pract.

[29] Casey S, Lanting S, Oldmeadow C, Chuter V (2019). The reliability of the ankle brachial index: a systematic review. J Foot Ankle Res.

[30] Gunnarsson T, Lindgren H, Gottsäter A, Pärsson H (2021). Intraprocedural transcutaneous oxygen pressure and systolic toe pressure measurements during and after endovascular intervention in patients with chronic limb threatening ischaemia. Eur J Vasc Endovasc Surg.

[31] Aerden D, Massaad D, von Kemp K (2011). The ankle-brachial index and the diabetic foot: a troublesome marriage. Ann Vasc Surg.

[32] Hyun S, Forbang NI, Allison MA, Denenberg JO, Criqui MH, Ix JH (2014). Ankle-brachial index, toe-brachial index, and cardiovascular mortality in persons with and without diabetes mellitus. J Vasc Surg.

[33] Tehan PE, Santos D, Chuter VH (2016). A systematic review of the sensitivity and specificity of the toe-brachial index for detecting peripheral artery disease. Vasc Med.

[34] Garcia LA (2006). Epidemiology and pathophysiology of lower extremity peripheral arterial disease. J Endovasc Ther.

[35] Taylor GI, Palmer JH (1987). The vascular territories (angiosomes) of the body: experimental study and clinical applications. Br J Plast Surg.

[36] van den Berg JC (2018). Angiosome perfusion of the foot: an old theory or a new issue?. Semin Vasc Surg.

[37] Fujii M, Terashi H (2019). Angiosome and tissue healing. Ann Vasc Dis.

